# Supplementary material for: Immobilized covalent triazine frameworks films as effective photocatalysts for hydrogen evolution reaction
Source: Nat Commun. 2021 Nov 15;12:6596. doi: 10.1038/s41467-021-26817-4 (PMC8593010; doi:10.1038/s41467-021-26817-4)
Supplement: Supplementary file 1 — Supplementary Information [file 41467_2021_26817_MOESM1_ESM.pdf]

Supporting Information

Immobilized Covalent Triazine Frameworks Films as Effective Photocatalysts for Hydrogen Evolution Reaction – Hu et al.

Section I. Materials and Methods

1. Materials

Benzene-1, 4-dicarbonitrile, N, N-dimethylformamide (DMF), cesium carbonate (Cs_2CO_3), tetrahydrofuran (THF), and absolute ethanol were of analytical grade and purchased from National Medicines Corporation Ltd. of China. Dimethyl sulfoxide (DMSO) of 99.7% purity, extra dried with molecular sieves with water content ≤ 50 ppm (by K.F.), was purchased from Energy Chemical Corporation Ltd. of China. 1, 4-phthalaldehyde was also of analytical grade and purchased from TCI. n-hexylamine was purchased from Energy Chemical Corporation Ltd. of China. All reagents were used without further purification. The synthesis procedure of terephthalamidine dihydrochloride was adopted from the reported literature [1].

Synthesis of terephthalamidine dihydrochloride [1]: To a solution of benzene-1,4-dicarbonitrile (1.28 g, 10.0 mmol) in 20 mLTHF, 40 mL of 1 M $LiN(SiMe_3)_2$ solution was added dropwise in 30 min at 0 °C. The mixture was stirred at 25 °C for 3 h and then cooled to 0 °C. The reaction was quenched by careful addition of 6 M HCl–EtOH (40 mL) and the mixture was set aside overnight. The precipitate was then filtered, washed with Et_2O, and then the powder was recrystallized from H_2O–EtOH mixture. Yield: (2.2 g, 93%). ^{1}H NMR (400MHz, DMSOd$_6$): δ = 9.63 (s, 4H, NH), 9.37 (s, 4H, NH), 8.03 (s, 4H, aromatic H).

2. Characterizations

The formation of triazine unit was confirmed by Fourier-transformed infrared (FT-IR) spectroscopy in ATR mode using a Bruker Vertex 70 FT-IR spectrometer, solid-state

^{13}C CP/MAS NMR spectroscopy using a WB 400 MHz Bruker Avance II spectrometer with the contact time of 2 ms (ramp 100) and pulse delay of 3 s, and X-ray photoelectron spectroscopy (XPS) on an Axis Ultra DLD 600 W instrument (Shimadzu, Japan). The morphology of CTFs was characterized by transmission electron microscopy (TEM) (Tecnai G2 F30, FEI Holland), a FEI Sirion 200 field emission scanning electron microscope (FE-SEM) and atomic force microscopy (AFM) using scanning probe microscopy SPM-9700 instrument (Shimadzu Japan). The film was transferred to Si/SiO_2 wafer without ultrasonic treatment for the thickness analysis measured by AFM.

Small angle X-ray scattering (SAXS)/ wide-angle X-ray scattering (WAXS) in transmission mode

Small angle X-ray scattering (SAXS) experiments were conducted on the Xeuss 2.0 system (Xenocs, France) with X-rays of wavelength of 1.54189 Å. The d-spacing values were calculated by using the formula $d = 2\pi/q$. The films were ground to powders and SAXS experiments were carried out in transmission mode.

Small angle X-ray scattering (SAXS)/wide-angle X-ray scattering (WAXS) experiments were performed at the beamline BL19U2 in shanghai synchrotron radiation facility (SSRF). The 1.0332 Å wavelength and the 2D detector Pilatus 300k was used for collecting the data. The 2D scattering data were analyzed using the software RAW 2.1.1 and pyFAI. The d-spacing values were calculated by using the formula $d = 2\pi/q$. The films were ground to powders and SAXS/ WAXS experiments were carried out in transmission mode. Silver behenate (AgBH) reference was used for the calibration of sample-detector distance.

Grazing incidence wide angle X-ray scattering (GIWAXS)

Grazing incidence wide angle X-ray scattering (GIWAXS) measurements were conducted on the Xeuss 2.0 system (Xenocs, France) with X-rays of wavelength of 1.54189 Å. The film samples were irradiated at a fixed angle of 0.15°. All samples were transferred onto silicon substrates before measurements.

Powder X-ray diffraction (PXRD)

Powder X-ray diffraction (PXRD) data were collected by a Smart X-ray diffractometer (Smartlab SE, Rigaku, Japan) with Cu Kα radiation (λ = 1.54178 Å). Films were ground to powders and mounted as integral films onto a silicon zero background holder. The PXRD patterns were recorded from 5 to 40° (2θ) with a step size of 0.02° and a scan rate of 5° per minute.

Nitrogen sorption analyses

Surface areas and pore size distributions were deduced from N_2 adsorption and desorption profiles using a ASAP 2460 analyzer at 77 K after the pretreatment of the samples by heating under vacuum at 120 °C for 12 h. The pore size distributions were calculated using the non-local density functional theory (NLDFT).

Electrochemical Measurements

Electrochemical measurement was performed using three electrode systems on a CHI 760E electrochemical workstation in 0.1 M KOH solution. A carbon rod and Saturated Calomel Electrode (SCE) was used as a counter and reference electrode, respectively. To prepare the working electrode, film after photocatalysis experiment was transferred to ITO support and 20 µL 5 wt.% Nafion solution was used as a binder followed by air-drying. The exposed geometric surface area was 1.0 cm^2. All the voltage values were

converted into reversible hydrogen electrode (RHE) (E vs VRHE = E(SCE) + 0.0591*pH + 0.2412). Linear sweep voltammetry (LSV) was performed at a scan rate of 5 mV s^{-1}, and the potential range from -0.5 ~ 0 V vs RHE.

The analysis of product of electrolysis:

A home-made sealed three electrode electrochemical cell was used and purged with N_2 gas for 10 min. The produced H_2 gas was analyzed by gas chromatography (GC) using a thermal conductivity detector (TCD). The carrier gas for TCD was N_2. The H_2 gas was purged out by using syringe of 3 mL from the cell and injected into GC.

Faradaic Efficiency: For the Faradaic efficiency (FE), GC data for H_2 evolution was collected during the 8500 s at constant voltage of (-0.35 V vs RHE) and FE was calculated using the following relationship,

$$\mathrm{FE} = \frac{2\mathrm{F.\,nH_2}}{\mathrm{Q}}$$

nH_2 is the amount of hydrogen generated, Q is the total amount of charge passed through the cell (C), and F is the faraday constant.

Apparent quantum yield (AQY)

The AQY was measured using the following conditions: 100.0 mL of TEOA aqueous solution (10 vol %, v/v), 25 °C, and band pass filters (420 nm, 435 nm, 450 nm, 475 nm, 500 nm). The light intensity at 420 nm, 435 nm, 450 nm, 475 nm and 500 nm was 31.8, 22.3, 19.1, 15.9, 15.9 mW cm^{-2}, respectively (Supplementary Table 6). The final AQY at a certain light irradiation wavelength was calculated according to the equation.

$$\mathrm{AQY}(\%) = \frac{2CN_A}{SPt\lambda/hc}\times 100\%$$

where C is the H_2 production amount (μmol) per hour; N_A is the Avogadro constant (6.02×10^{23}/mol), h is the Plank constant (6.626×10^{-34} J/s), c is vacuum light velocity (3×10^8 m/s), λ is the monochromatic light wavelength (nm), t is the light irradiation time (1 h) and P is the incident monochromatic light intensity (W). The photocatalytic rates are not proportional to the photocatalyst mass because light absorption reaches saturation at some point. AQY should accordingly be measured when the amount of photocatalyst is sufficient and the incident light is effectively absorbed by the photocatalysts. The equation does not include the parameter: catalyst amount, which means the AQY value would be vary with catalyst amount.

Optimization of the reaction temperature

The effect of temperature was investigated. The uniform film without any visible bumps or aggregates on the surface was kept for 3 days at 70 ^oC and about 1 day at 90 ^oC. It should be mentioned that even at 180 °C, 12 h time was required to produce uniform layers (when the temperature was 120 °C, the evaporation rate of DMSO was too fast, so the reaction was carried out in a hydrothermal reactor). The morphology of the CTF film obtained at 70 ~ 180 °C was the same but a longer time was required at lower temperature and a special device was used at higher temperature. These control experiments showed similar results as reported by Feng et al., that reaction time depends on the temperature, which determines the diffusion rates of monomers. Based on the above discussions the reaction temperature was chosen to be 100 °C. Besides, some special devices with better gas tightness were also used.

Section II. Supplementary Figures and Tables

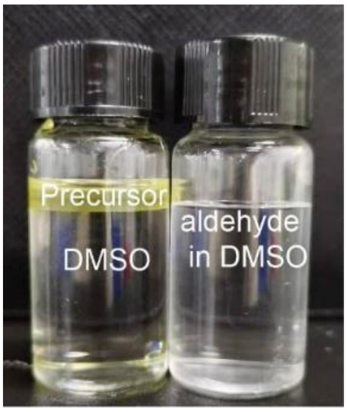

Supplementary Figure 1. Photographic image of the imine precursor and aldehyde monomer in DMSO. Imine precursor floating on DMSO (left); and aldehyde monomer dissolved in DMSO.

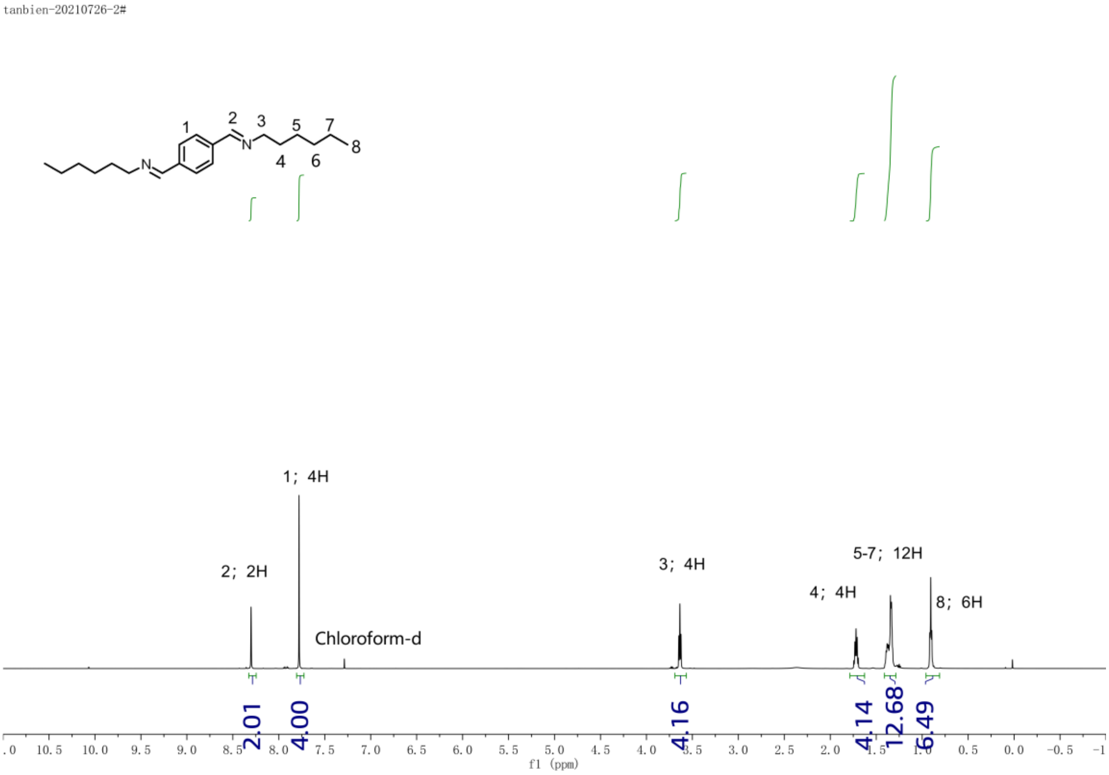

Supplementary Figure 2. ^{1}H-NMR spectra of imine precursor in d-chloroform.

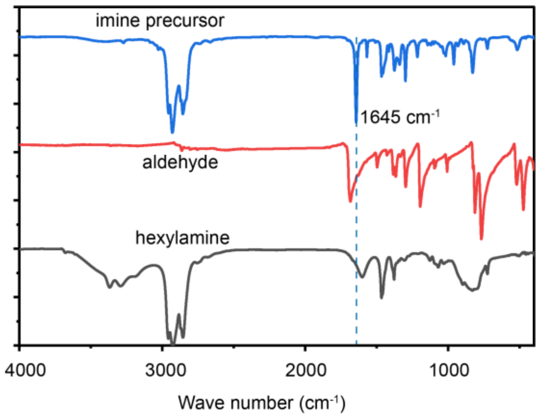

Supplementary Figure 3. FT-IR spectra of imine precursor, aldehyde and hexylamine.

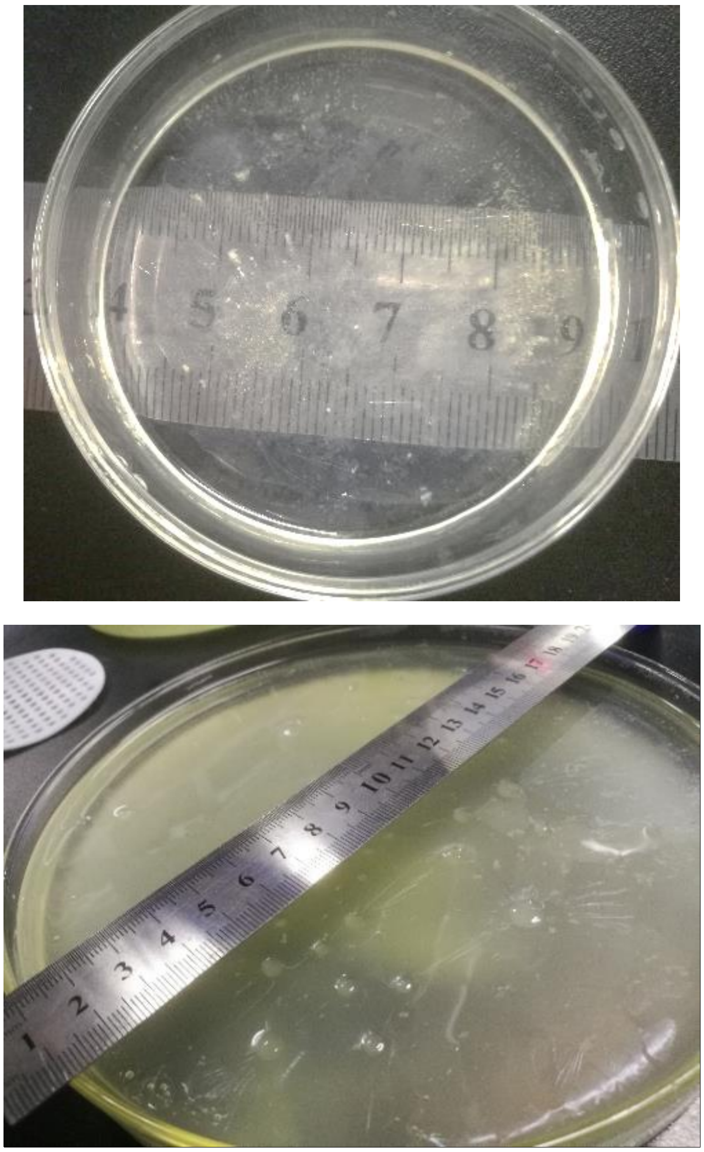

Supplementary Figure 4. The film on the DMSO surface with the different lateral size that was limited by the reactor dimensions.

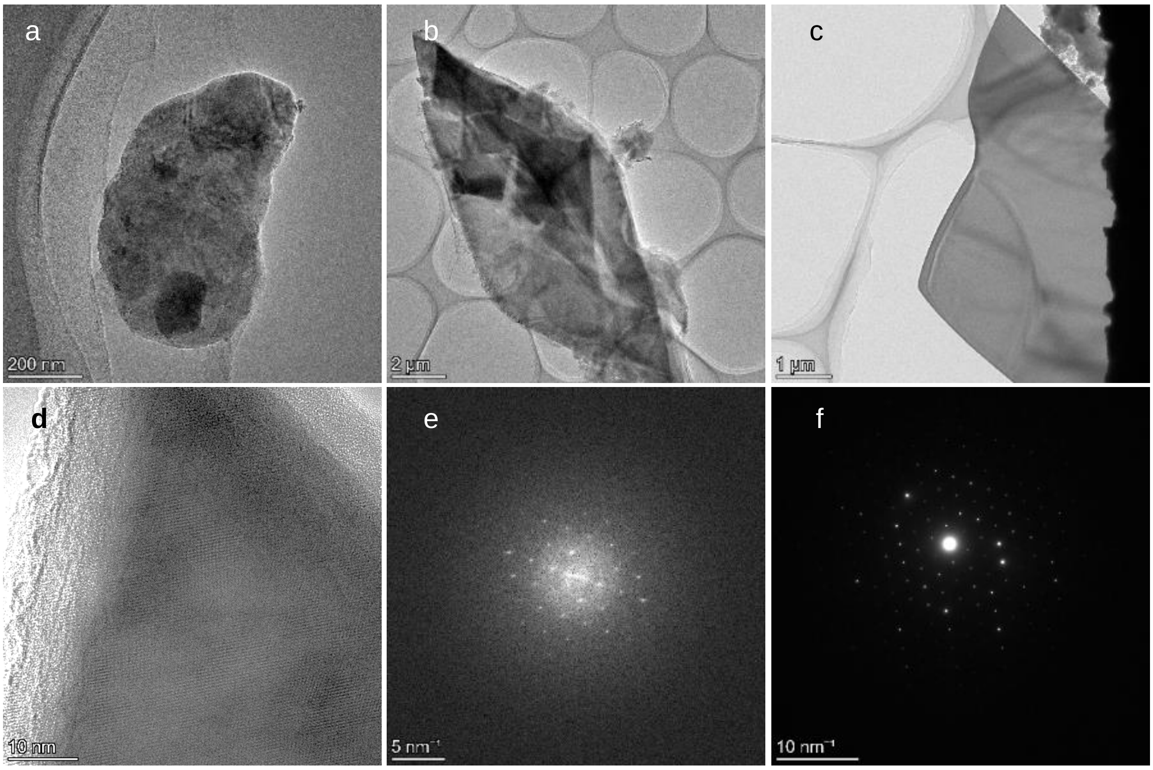

Supplementary Figure 5. TEM images of film dispersed in EtOH by ultra-sonication. **a** – **c** TEM images of film at different places. **d** Clear lattice fringe shown at higher magnification. **e** FFT image and (**f**) SAED of piece showed in Supplementary **Figure 5c**.

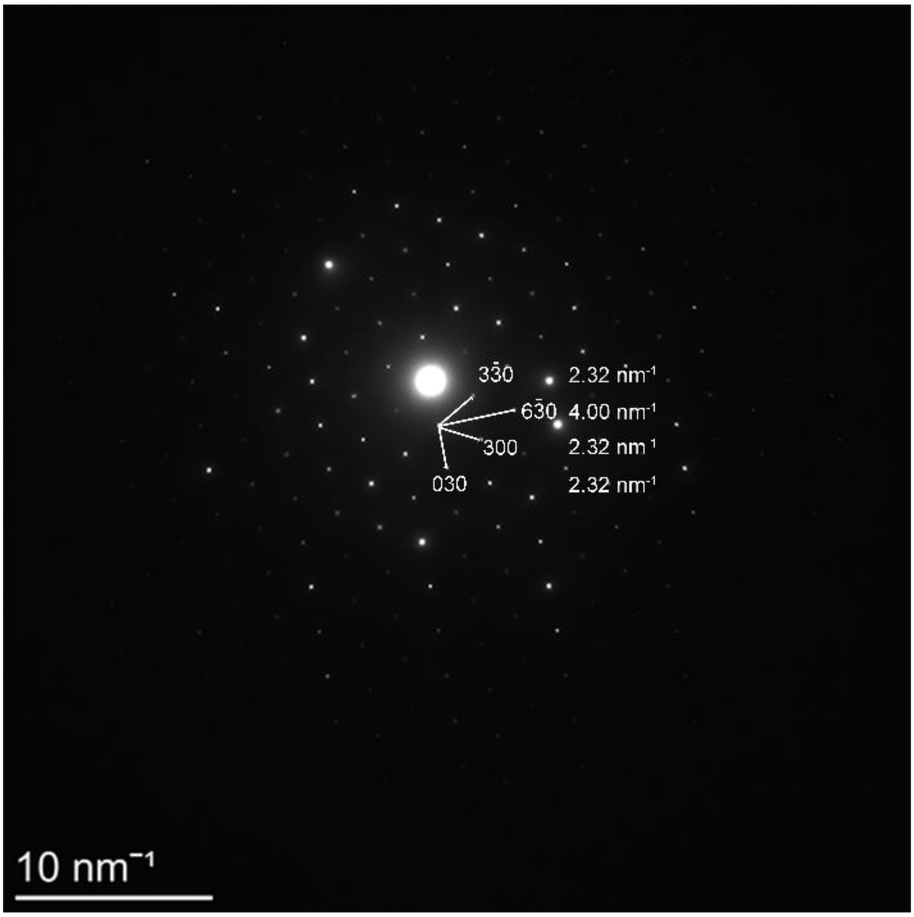

Supplementary Figure 6. SAED pattern of a single-crystalline domain along [001] direction.

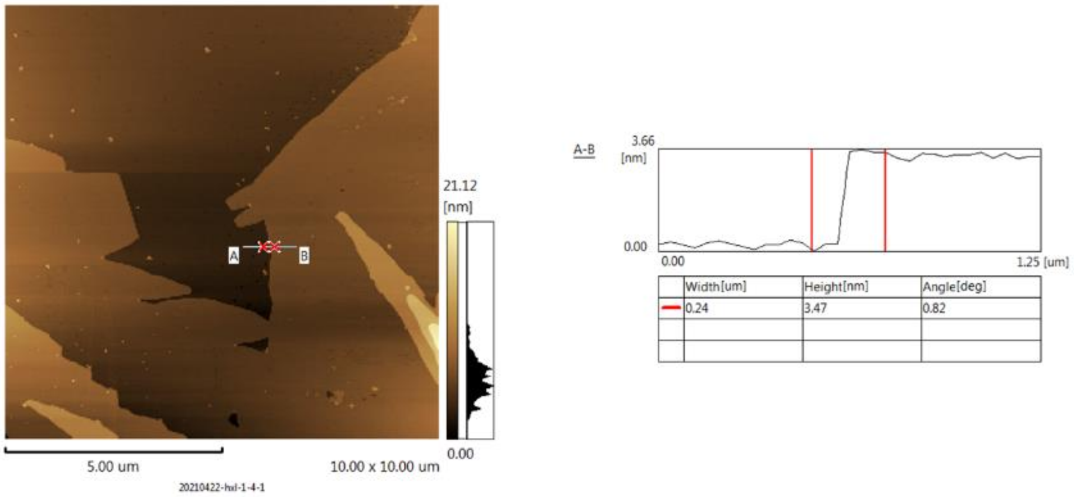

Supplementary Figure 7. AFM image of CTF nanosheets after dispersing the film in ethanol by ultrasonication.

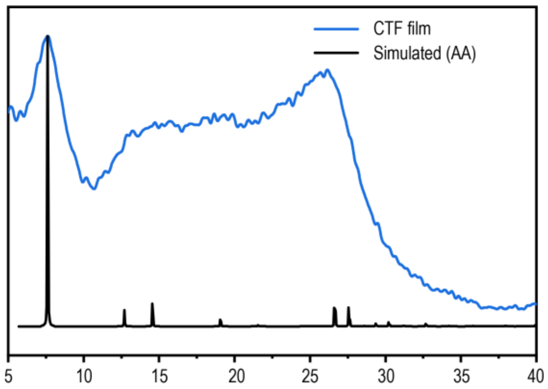

Supplementary Figure 8. PXRD of collected CTF film and its simulated XRD pattern with AA stacking.

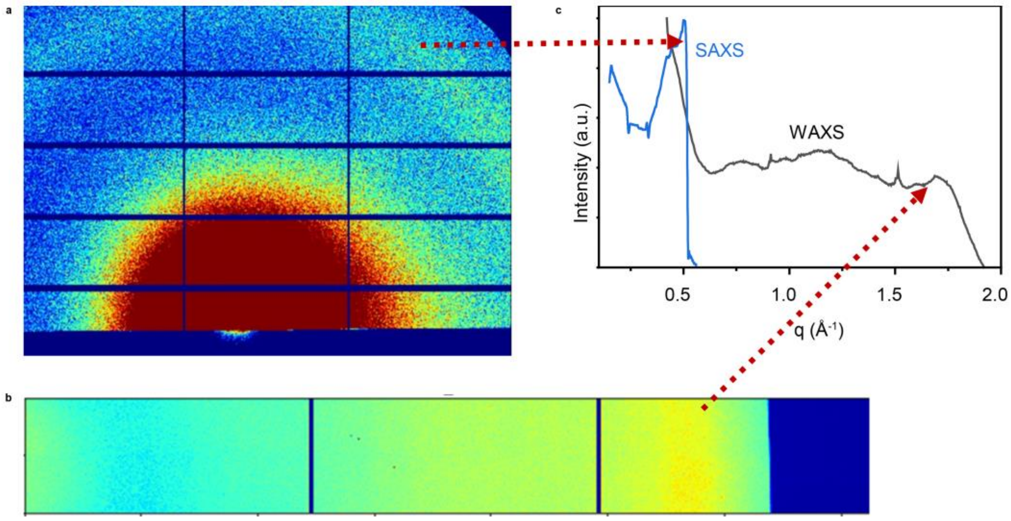

Supplementary Figure 9. Synchrotron SAXS/WAXS measurement of the film. **a** SAXS image. **b** WAXS image. **c** Integration intensity profiles of SAXS/WAXS.

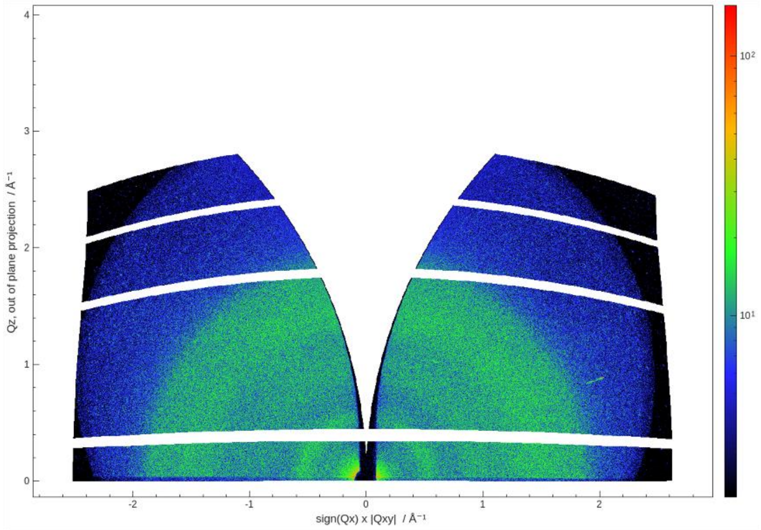

Supplementary Figure 10. GIWAXS measurement of the film.

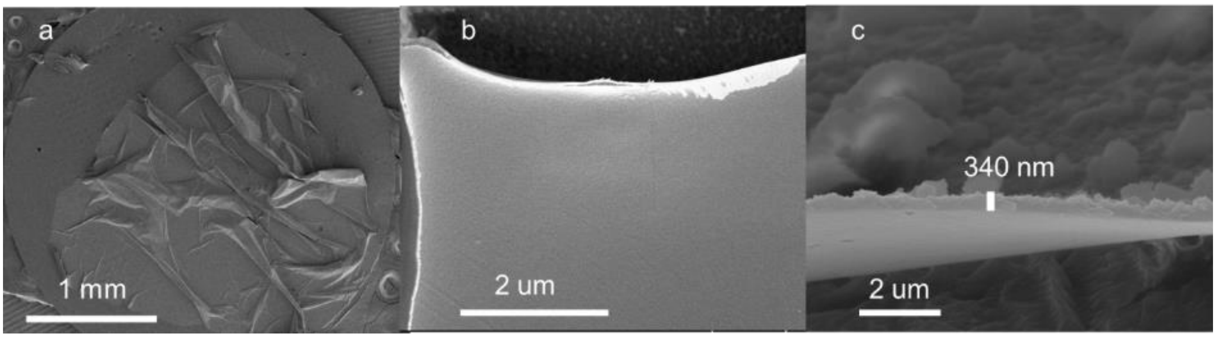

Supplementary Figure 11. (**a** - **b**) SEM images of CTF film (340 nm thick) at low magnification. **c** Cross-sectional SEM images.

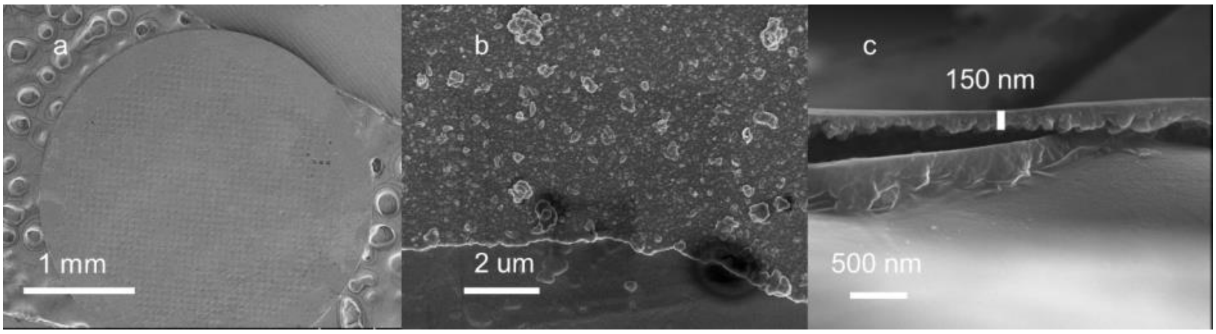

Supplementary Figure 12. (**a** - **b**) SEM images of CTF film (150 nm thick) at low magnification. **c** Cross-sectional SEM images.

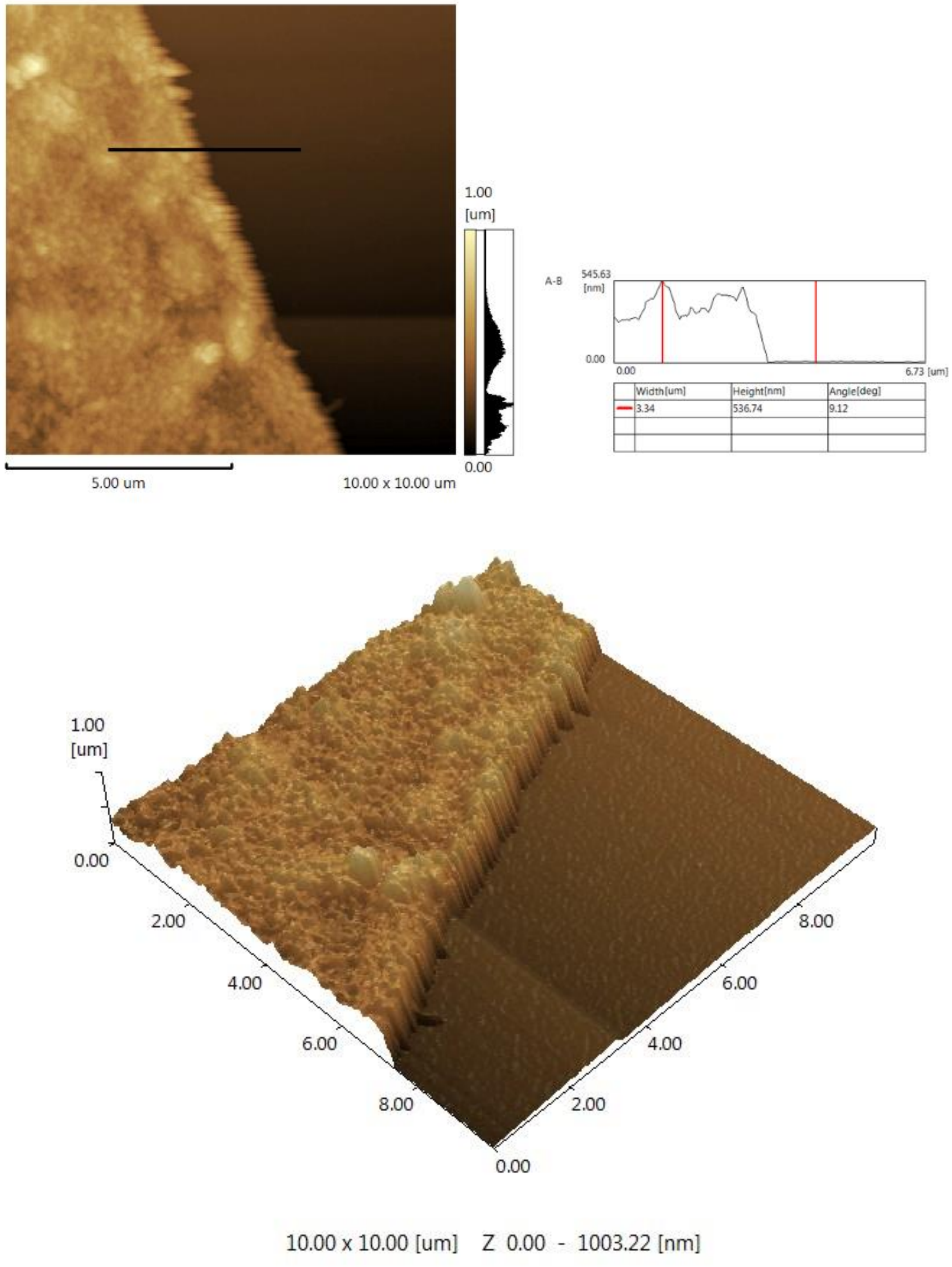

Supplementary Figure 13. AFM image of CTF film with a thickness of 540 nm.

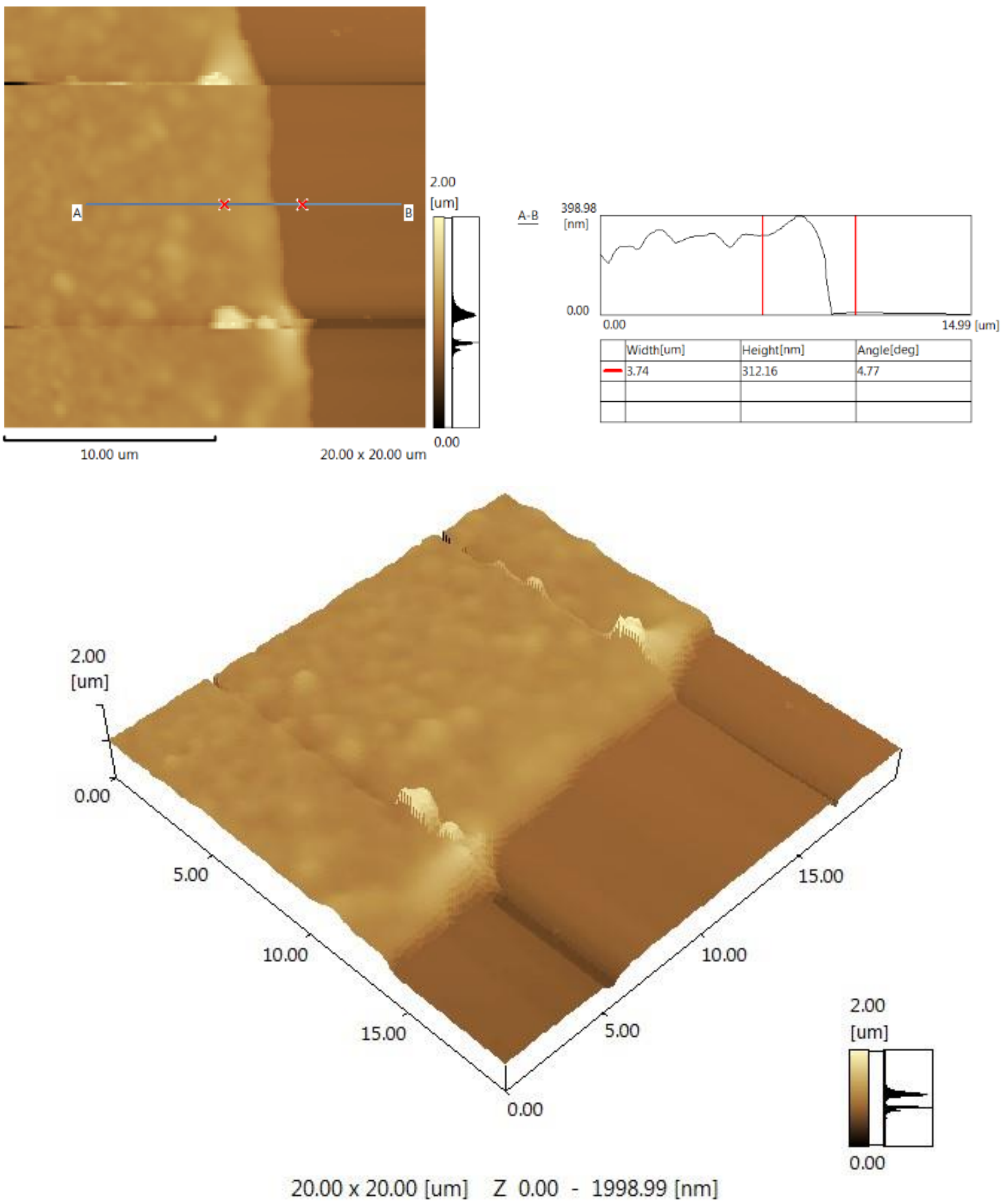

Supplementary Figure 14. AFM image of CTF film with a thickness of 300 nm.

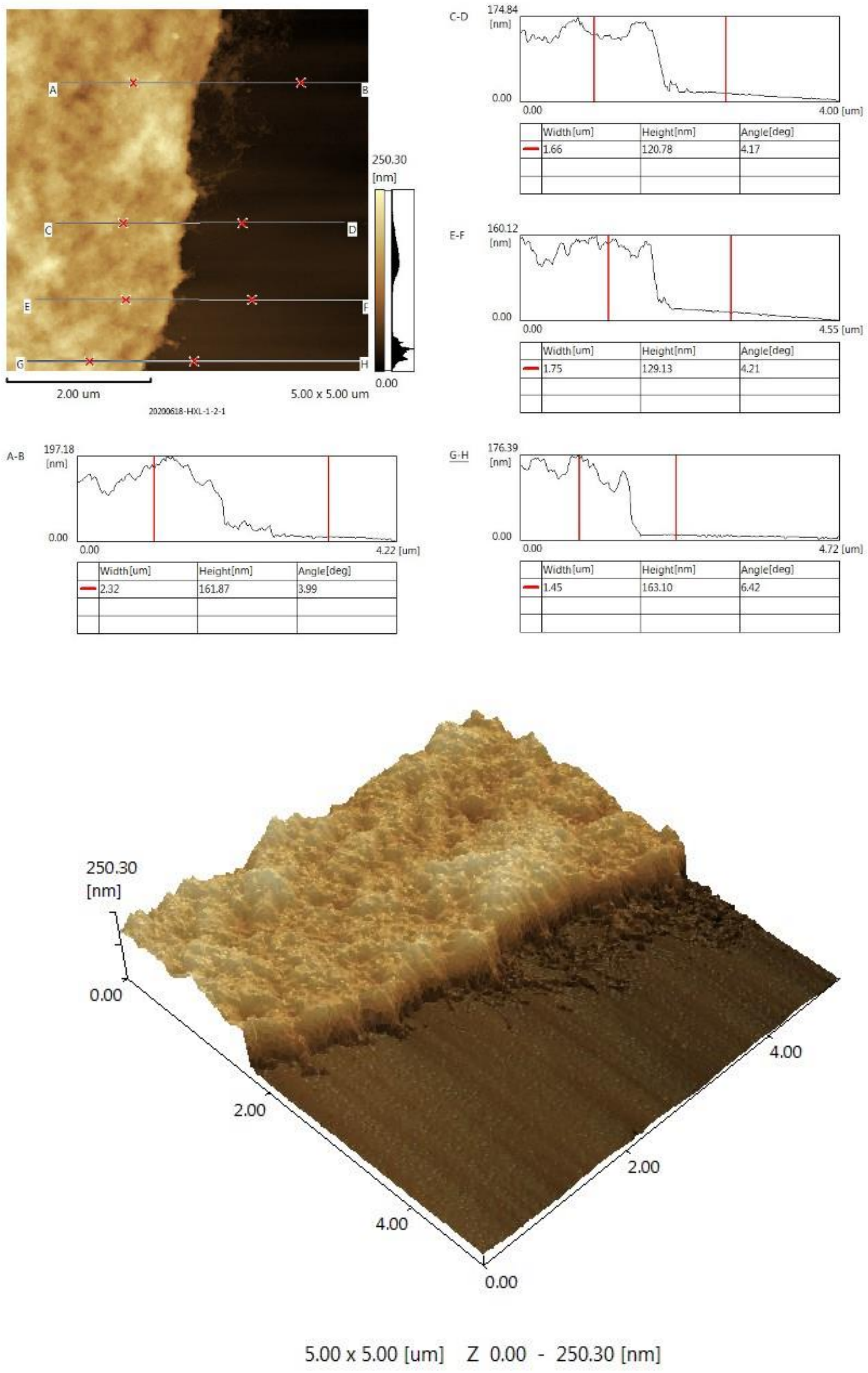

Supplementary Figure 15. AFM image of CTF film with a thickness of 130 nm.

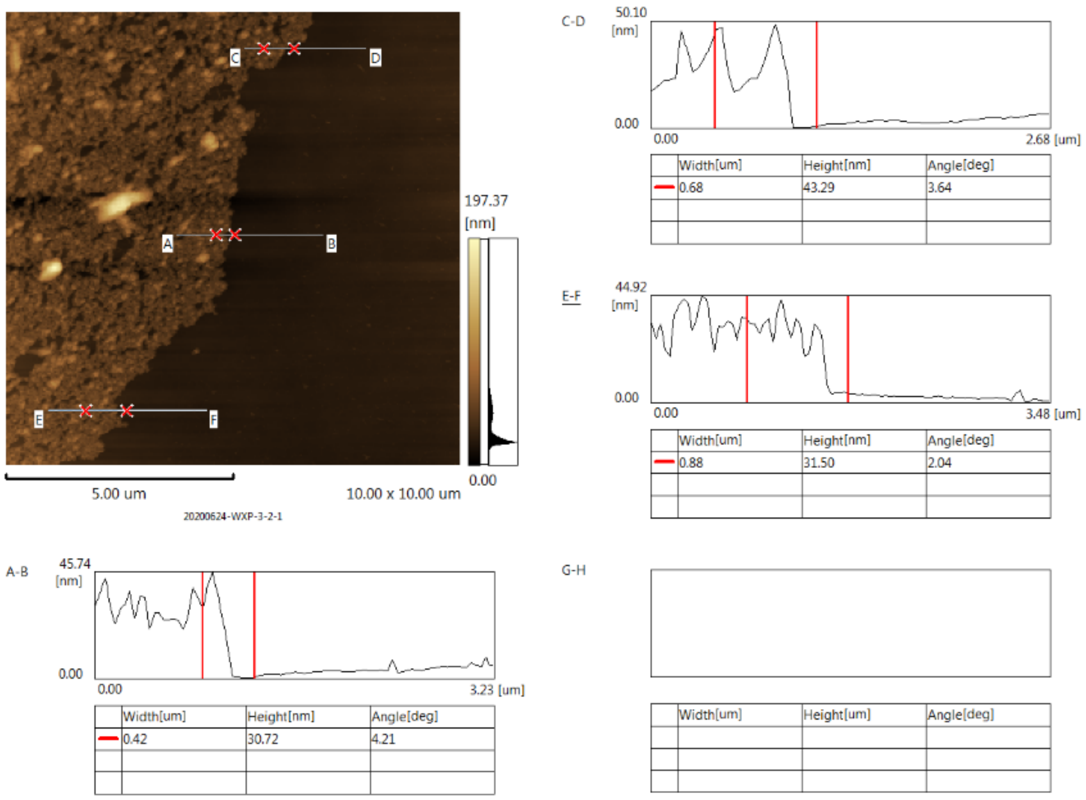

Supplementary Figure 16. AFM image of CTF film with a thickness of 30 nm.

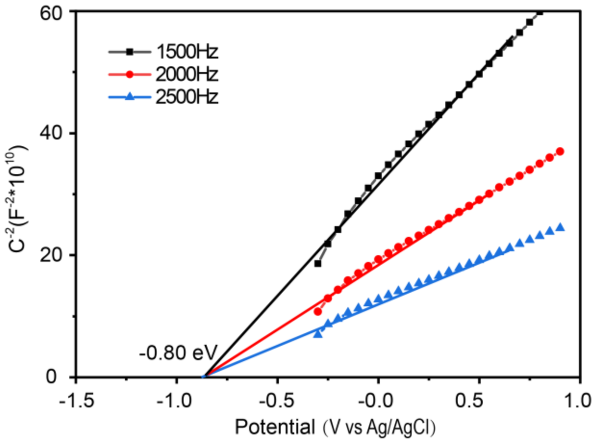

Supplementary Figure 17. Mott-Schottky plots of CTF film (500 nm).

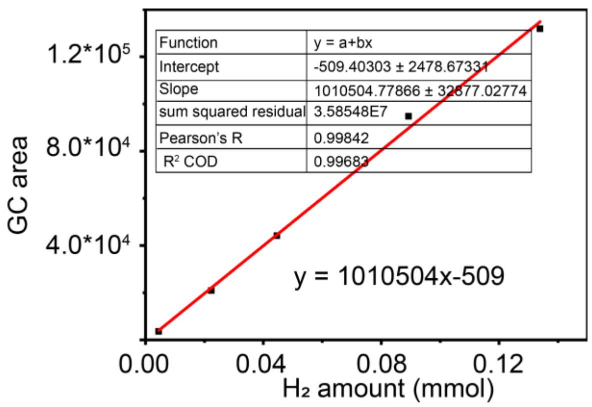

Supplementary Figure 18. Calibration curves for the quantification of produced H_2 using gas chromatography (GC) and TCD detector.

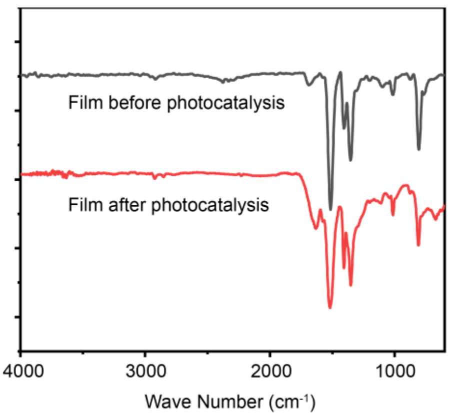

Supplementary Figure 19. FT-IR spectra of CTF film before and after photocatalytic experiment.

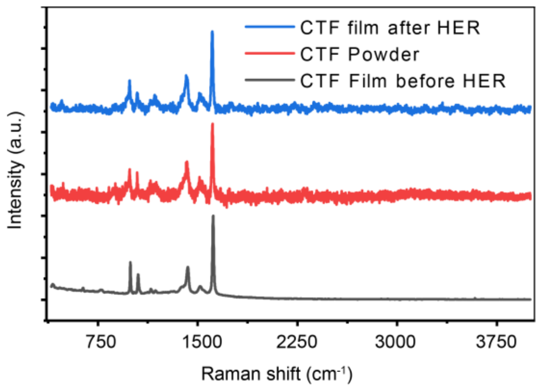

Supplementary Figure 20. Raman spectra of CTF film before and after photo catalytic experiment.

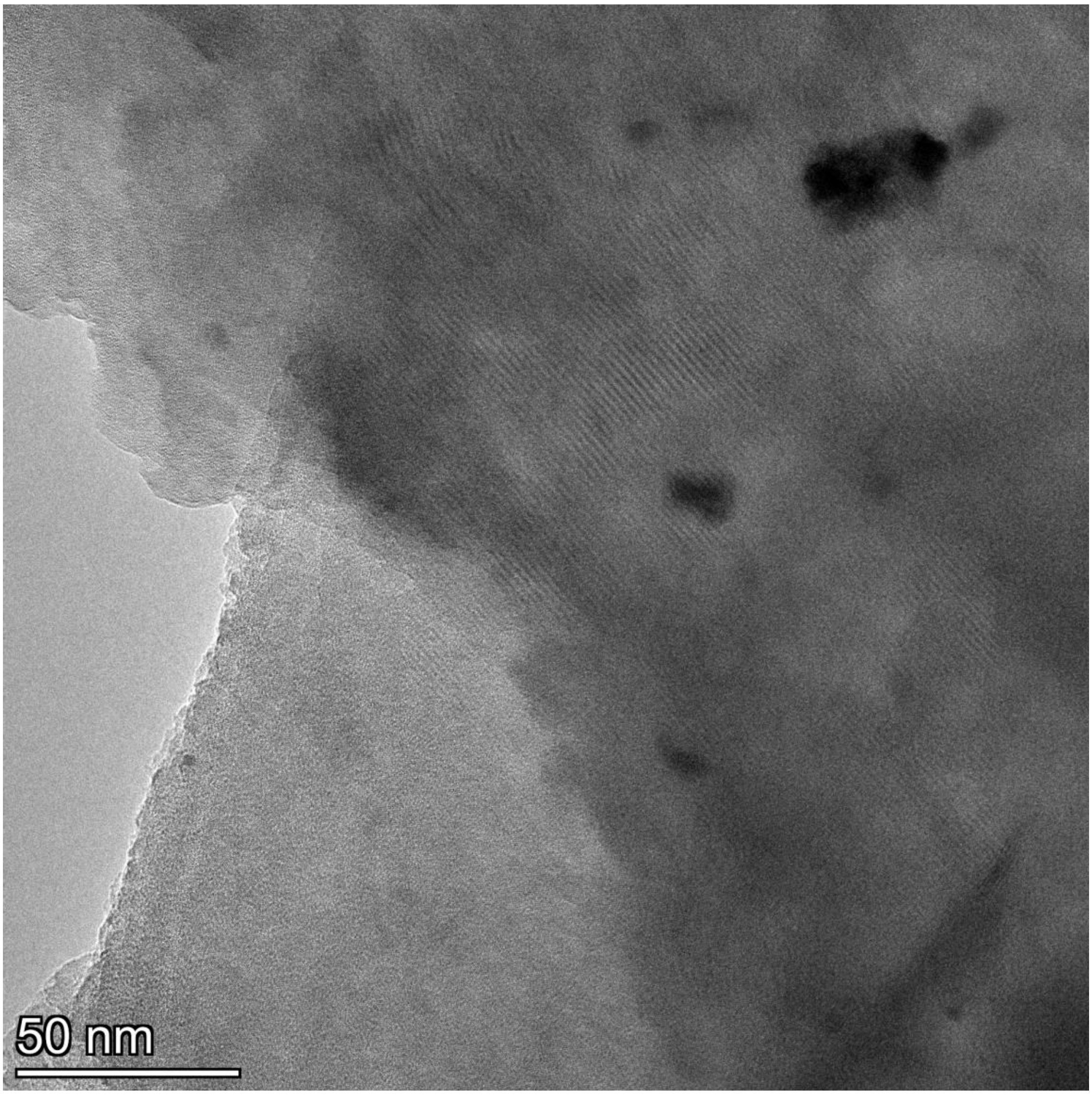

Supplementary Figure 21. TEM images of CTF film after photocatalysis. Bright field images. The broader fringes are Moire fringes.

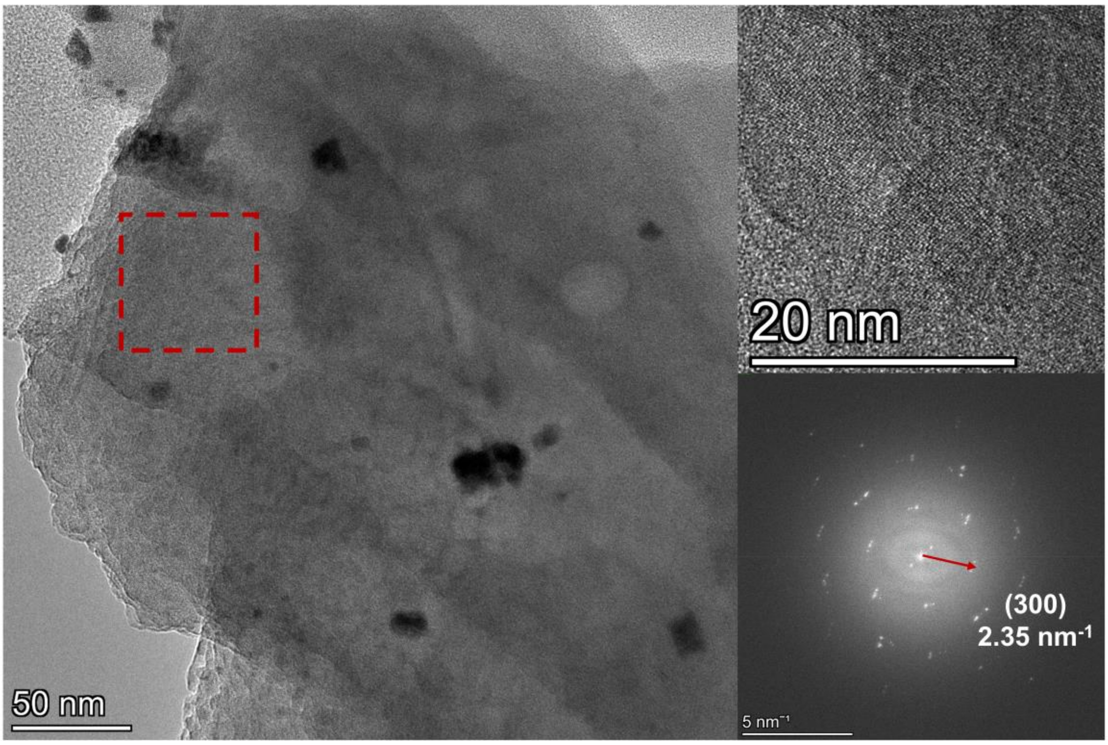

Supplementary Figure 22. TEM images of CTF film after photocatalysis. Bright field images and FFT images. Zone axis [001].

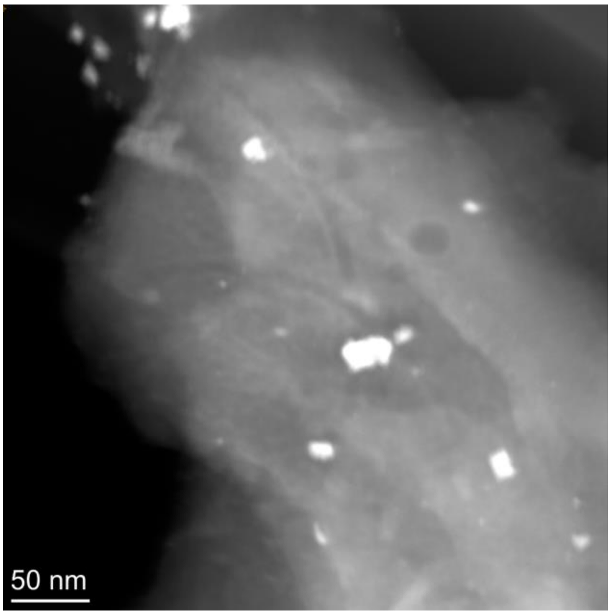

Supplementary Figure 23. TEM dark field images of CTF film after photocatalysis that shown in Supplementary Figure 21 - 22.

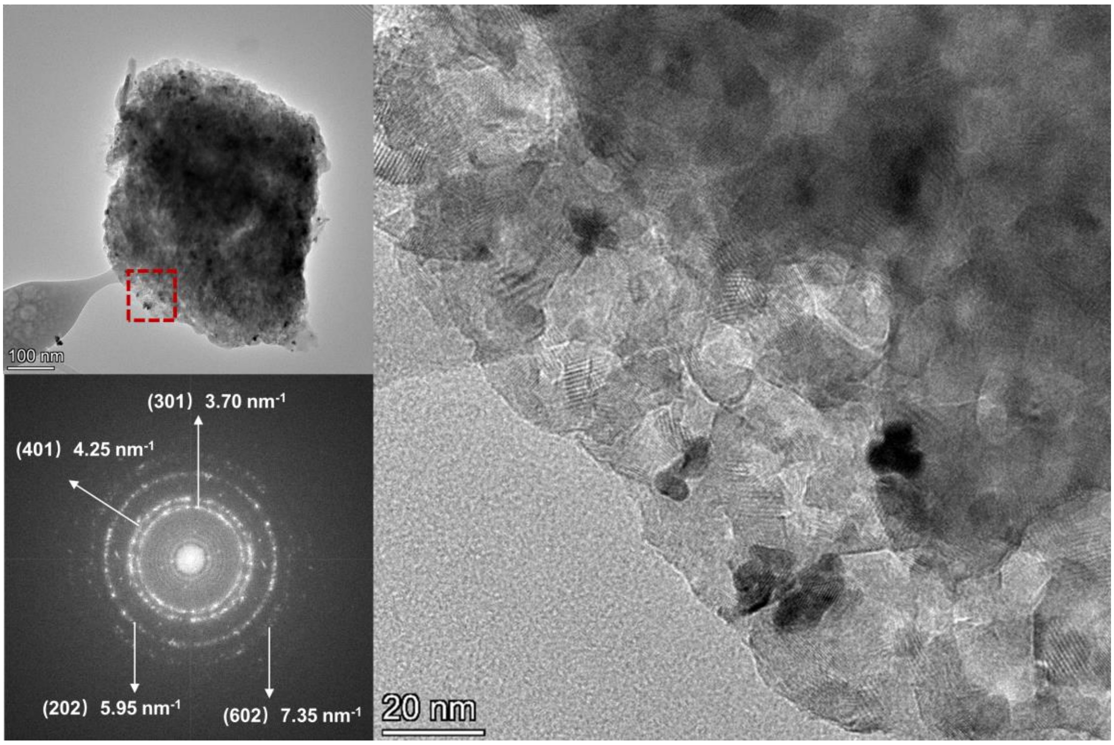

Supplementary Figure 24. TEM images of CTF film after photocatalysis. Bright field images and FFT images. Zone axis [010].

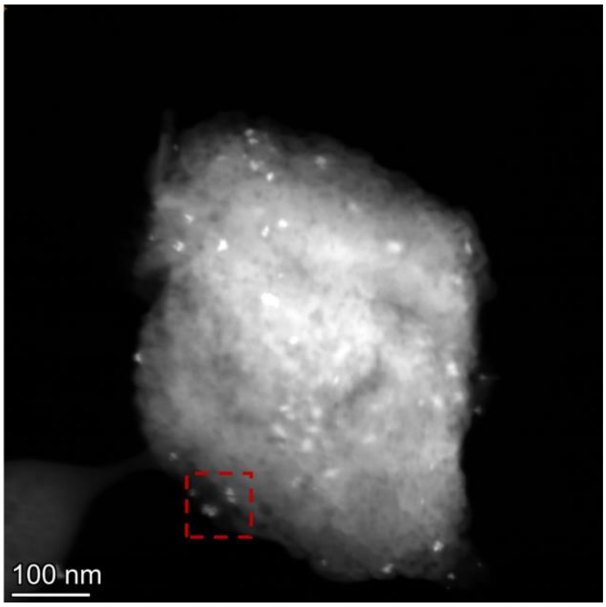

Supplementary Figure 25. TEM dark field images of CTF film after photocatalysis shown in Supplementary Figure 24.

Supplementary Table 1. Lattice parameters obtained by DFT calculation, SAED and GIWAXS measurements.

structure	a	b	c	α	β	γ
CTF1 (DFT) [1]	14.5	14.5	3.6	90	90	120
SAED	14.9	14.9	N.A	N.A	N.A	120
GIWAXS	14.0	14.0	3.5	N.A	N.A	N.A

Supplementary Table 2. Calculation details of GIWAXS.

q ($Å^{-1}$)	hkl	$d_{(hkl)}$ *vs.* q	$d_{(hkl)}$ *vs.* ɑ	ɑ
0.51	100	d = 2Π/q	d = √3/2ɑ	14.0
0.9	110	d = 2Π/q	d = 1/2ɑ	14.0
1.0	200	d = 2Π/q	d = √3/4ɑ	14.5

Supplementary Table 3. EA results of the film.

Methods	N(%)	C(%)	H(%)
Theoretical	21.9	75.0	3.1
EA (before HER)	17.3	68.6	4.7
XPS (before HER)	9.7	74.7	n.a
EDS (after HER)	13.0	73.0	n.a

Supplementary Table 4. Photocatalytic H_2 evolution performance of the reported COF photocatalysts (powder type).

Photocatalyst	Catalyst amount	$r(H_2)$ mmol g^{-1} h^{-1}	Co-catalyst	AQY (%)	Reference
FS-COF	5 mg	10.1	3 wt% H_2PtCl_6	3.2 (λ = 420 nm)	[3]
N_3-COF	10 mg	1.7	0.68 wt% Pt	0.44 (λ = 450 nm) 0.17 (λ = 400 nm)	[4]
TFPT-COF	10 mg	1.97	2.2 wt% Pt	2.2 (λ = 400 nm)	[5]
TP-BDDA	10 mg	0.32	3 wt% Pt	1.3 (λ = 420 nm)	[6]
g-$C_{40}N_3$-COF	50 mg	2.60	3 wt% Pt	4.84 (λ = 420 nm)	[7]
CTF- C1	50 mg	5.1	3 wt% Pt	n.a	[8]
CTF-HUST-1	50 mg	1.46	3 wt% Pt	n.a	[9]
CTF-HUST-A1	50 mg	9.2	3 wt% Pt	n.a	[10]
CTF-Film	~ 1 mg	10.2	2 wt% Pt	0.11 (λ = 420 nm)	This work

Supplementary Table 5. Photocatalytic H_2 evolution performance of the reported film based system.

	$r(H_2)$	Light	Sacrifical agent	Thickness	Method	Reference
**immobilized Pt@mp-CN lab reactor**	0.19 L m^{-2} h^{-1}	solar simulator 1000 W m^{-2}	TEOA	~ 8 µm	casting	[2]
**immobilized Pt@FS-COF**	15.8 mmol h^{-1} m^{-2} (0.36 L m^{-2} h^{-1})	AM1.5G	AA	n.a	casting	[3]
**CTF Film**	5.4 mmol m^{-2} h^{-1} (0.12 L m^{-2} h^{-1})	>420 nm 955 W m^{-2}	TEOA	500 nm	Direct film loading	This work

Supplementary Table 6. AQY of CTF film using five band pass filters.

λ	P (mW cm^{-2})	C (1 h)	AQY
420 nm	31.8	4.1 μmol	0.11%
435 nm	22.3	1.9 μmol	0.07%
450 nm	19.1	1.0 μmol	0.04%
475 nm	15.9	0.5 μmol	0.02%
500 nm	15.9	0.2 μmol	0.01%

Supplementary Table 7. Parameters used for the calculation of Faradaic Efficiency.

H_2 amount	Q
11.79 μmol	2.476 C

References

[1]. Wang, K. W. et al. Covalent Triazine Frameworks via a Low-Temperature Polycondensation Approach. *Angew. Chem. Int. Ed.* **56**, 14149-14153 (2017).

[2] Schröder, M. et al. Hydrogen Evolution Reaction in a Large-Scale Reactor using a Carbon Nitride Photocatalyst under Natural Sunlight Irradiation. *Energy Technol* **3**: 1014-1017 (2015).

[3] Wang, X. et al. Sulfone-containing covalent organic frameworks for photoc atalytic hydrogen evolution from water. *Nat. Chem.* **10,** 1180-1189 (2018).

[4] Vyas, V. S. et al. A tunable azine covalent organic framework platform for visible light-induced hydrogen generation. *Nat. Commun.* **6**, 8508 (2015).

[5] Stegbauer, L., Schwinghammer, K., Lotsch, B. V. A hydrazone-based coval ent organic framework for photocatalytic hydrogen production. *Chem. Sci.* **5**, 27 89-2793 (2014).

[6] Pradip Pachfule et al. Diacetylene Functionalized Covalent Organic Framework (COF) for Photocatalytic Hydrogen Generation. *J. Am. Chem. Soc.* **140**, 1423–1427 (2018).
[7] Bi, S. et al. Two-dimensional semiconducting covalent organic frameworks via condensation at arylmethyl carbon atoms. *Nat. Commun.* **10**, 2467 (2019).
[8] Liu, M. Y., et al. Crystalline Covalent Triazine Frameworks by In Situ Oxidation of Alcohols to Aldehyde Monomers. *Angew. Chem. Int. Ed.* **57**, 11968-11972 (2018).
[9] Wang, K. W. et al. Covalent Triazine Frameworks via a Low-Temperature Polycondensation Approach. *Angew. Chem. Int. Ed.* **56**, 14149-14153 (2017).
[10] Zhang, S. Q., Cheng, G., Guo, L. P., Wang, N., Tan, B. E., Jin, S. B. Strong-Base-Assisted Synthesis of a Crystalline Covalent Triazine Framework with High Hydrophilicity via Benzylamine Monomer for Photocatalytic Water Splitting. *Angew. Chem. Int. Ed.* **59**, 6007-6014 (2020).
