# Supplementary material for: Immobilized covalent triazine frameworks films as effective photocatalysts for hydrogen evolution reaction
Source: Nat Commun. 2021 Nov 15;12:6596. doi: 10.1038/s41467-021-26817-4 (PMC8593010; doi:10.1038/s41467-021-26817-4)
Supplement: Supplementary file 3 — Description of Additional Supplementary Files [file 41467_2021_26817_MOESM3_ESM.pdf]

File Name: Supplementary Movie  1

Description: The movie of the CTF film immersed in ethanol are provided.
